# Treatment against helminths in Norwegian sheep: a questionnaire-based survey

**DOI:** 10.1051/parasite/2021061

**Published:** 2021-09-01

**Authors:** Maiken Gravdal, Lucy J. Robertson, Kristoffer R. Tysnes, Johan Höglund, Christophe Chartier, Snorre Stuen

**Affiliations:** 1 Institute for Production Animal Clinical Science, Faculty of Veterinary Medicine, Norwegian University of Life Sciences Sandnes 4325 Norway; 2 Swedish University of Agricultural Sciences, Department of Veterinary Public Health, Section for Parasitology P.O. Box 7036 Uppsala Sweden; 3 BIOEPAR, INRAE Oniris 44307 Nantes France

**Keywords:** Anthelmintic resistance, Sheep, Questionnaire survey, Parasite control, Liver fluke, Gastrointestinal nematodes

## Abstract

A questionnaire was distributed to 5487 farmers throughout Norway in order to obtain information about management practices regarding helminth infections in sheep. In addition, the farmers’ perceptions of helminths and anthelmintic efficacy were investigated. Most farmers (80%) treated prophylactically against nematodes, and 24% also used prophylactic treatment against *Fasciola hepatica*. Overall, few farmers (11%) used parasitological analysis as a tool to assess the timing of treatment, but rather based it on other factors such as previous experience (70%). In the surveyed sheep flocks, the use of benzimidazoles was reduced from 2018 (52%) to 2019 (47%) (*p* < 0.01), whereas the use of macrocyclic lactones increased from 2017 (23%) to 2019 (36%) (*p* < 0.001). Poor anthelmintic efficacy was suspected by 10% of the farmers, and 11% reported that helminths were an increasing problem in their flocks. The majority of farmers (72%) considered their veterinarian as the most important advisor for treatment of parasites, but reported a high level of uncertainty regarding which parasites were present in their flocks, with unknown status most frequently reported for *Haemonchus contortus* (71.5%). This is probably related to the fact that very few farmers (15%) regularly test their animals for parasites. The present study provides up-to-date information on treatment practices for helminths in Norwegian sheep flocks.

## Abstract (fr)

Un questionnaire a été distribué à 5487 éleveurs dans l’ensemble de la Norvège afin d’obtenir des informations sur la gestion des helminthiases chez les ovins. Le questionnaire a porté, en outre, sur la perception des éleveurs concernant les helminthiases et l’efficacité des anthelminthiques. La plupart des éleveurs (80 %) réalisent des traitements prophylactiques contre les nématodes et 24 % font de même vis-à-vis de *Fasciola hepatica*. Globalement, peu d’éleveurs (11 %) recourent aux analyses parasitologiques pour déterminer les dates de traitement, s’appuyant plutôt sur d’autres éléments tels que leur propre expérience (70 %). Dans les troupeaux enquêtés, l’utilisation des benzimidazoles a diminué de 2018 (52 %) à 2019 (47 %) (*p* < 0,01) tandis que celle des lactones macrocycliques a augmenté entre 2017 (23 %) et 2019 (36 %) (*p* < 0,001). Une faible efficacité des anthelminthiques est suspectée par 10 % des éleveurs tandis que 11 % des éleveurs signalent que les helminthiases sont un problème croissant dans leurs troupeaux. La majorité des éleveurs (72 %) considèrent leur vétérinaire comme le conseiller le plus important en matière de traitement antiparasitaire mais rapportent un haut niveau d’incertitude concernant le type de parasites présents dans leur troupeau, en particulier en ce qui concerne la présence *d’Haemonchus contortus* (71,5 %). Ceci est probablement à relier au fait que très peu d’éleveurs (15 %) testent régulièrement leurs animaux vis-à-vis des parasites. La présente étude fournit des informations actualisées sur les pratiques de traitement anthelminthique des troupeaux ovins en Norvège.

## Introduction

Gastrointestinal nematodes (GINs) and the common liver fluke (*Fasciola hepatica*) are important helminths that can cause clinical and subclinical disease, as well as economic losses, in small ruminant production [8]. These infections have a crucial impact on animal welfare in the global ruminant livestock industry [31]. Anthelmintic treatment is the most common way of controlling helminth infections in ruminants. However, anthelmintic resistance (AR) is an emerging threat to the productivity and welfare of sheep in many parts of the world [26, 41], and is also recognized as a widespread and increasing challenge in Europe [4, 39]. This has a major economic impact due both to lost production, as well as costs of anthelmintic drugs that may not be effective [8]. Several risk factors that may enhance the development of AR have been identified. These include high frequency of treatment, using the dose-and-move strategy, absence of rotation between anthelmintic classes, introduction of animals carrying resistant parasites to the flock, under-dosing with anthelmintics, and blanket non-targeted treatments [6, 9, 10, 19, 28, 40, 42, 49, 51].

Following the increased reporting of AR [38], focus has been directed towards sustainable approaches for parasite control to slow down this development [50]. One of the main pillars to implementing sustainable parasite control is to base the treatment on diagnostics; namely informed treatment [7]. This gives the opportunity to decrease the use of anthelmintics, by targeting the treatment to specific animals or part of the flock based on the risk of developing ill-thrift. Targeted treatment (TT) is a concept for optimized treatment decision-making at the flock level based on a marker of infection, e.g., fecal egg count (FEC), while targeted selective treatment (TST) is based on treatment of individuals, both to preserve production and control infection [7, 27]. As a response to AR development of helminths in Europe, the CVMP (Committee for Medicinal Products for Veterinary Use) have recommended systematic monitoring programs, TST, and prescription-only status for anthelmintics used in food-producing animals [17]. It is important to assess and heighten the awareness among farmers, veterinarians, authorities, and the sheep industry at large regarding anthelmintic treatment routines and ensure that recommendations are followed up concerning minimizing the risk of AR development.

In Norway, anthelmintics must be prescribed by a veterinarian, but there is currently no systematic surveillance program. Two anthelmintic classes are licensed for use against GINs in sheep; benzimidazoles (BZ) and macrocyclic lactones (ML), of which BZ has been the dominating class for decades [13]. Albendazole (ABZ) is the only drug licensed for use against *F. hepatica* in Norway, while use of triclabendazole (TCBZ) requires approval from the Norwegian Medicines Agency (NMA). This means that the veterinarian must provide an application form to NMA, in order to justify the demand for TCBZ. There are few studies on the occurrence and prevalence of AR for GINs in Norway. BZ resistance has been detected in *Haemonchus contortus* in several sheep flocks in Norway by the fecal egg count reduction test (FECRT) [13], and later confirmed by experimental infections and controlled efficacy testing (CET) [16]. Resistance against ML has also been detected in *H. contortus* in one sheep flock in Norway by FECRT [35]. Moreover, lack of efficacy to BZ in *F. hepatica* has been reported in several European countries (e.g., [18, 33]), but there are currently no documented cases about this in Norway. Control practices against GINs in Norwegian sheep flocks were investigated in 2007, by a questionnaire survey among farmers from the northern, inland, and coastal areas [14]. The results suggested that, at that time, more than 90% of the sheep flocks in Norway could be at risk of under-dosing when administrating anthelmintics. Dissemination of information to farmers regarding the importance of quarantine routines, drench-gun calibration, avoiding under-dosing, and correct administration of the drug has been an area of focus in the Sheep Health Service [1, 21, 22], following the previous study by Domke et al. 2011 [14].

The aim of our study was to obtain information on the current situation regarding management of major helminth infections in Norwegian sheep flocks. A second objective was to acquire knowledge about Norwegian sheep farmers’ own perceptions regarding GINs and *F. hepatica*, as well as anthelmintic efficacy.

## Materials and methods

### Questionnaire

In February 2020, a questionnaire survey was distributed by email to 5487 farmers. These were all members of the Norwegian Sheep Recording System (NSRS) with a registered email address and represent approximately 40% of sheep farmers in Norway [43]. A Questback data-management system was used for the questionnaire, and farmers that did not respond were reminded after one week, and then again two weeks later. A pilot survey was tested on a limited number of sheep farmers in advance to ensure that the questions were understandable and to avoid misinterpretations. Before dispatch of the questionnaire, information about the survey was published online in a newsletter by Sheep Health Service (Animalia), encouraging sheep farmers to participate. Animalia is a health service company within the Norwegian meat and egg industry that provides various veterinary health services to farmers, including guidelines for parasite management in sheep. Around 3760 sheep farmers subscribe to their newsletter.

The questionnaire was divided into four sections, the first of which contained contact information and location, and the subsequent three sections focused on flock and parasitic infections (see Supplementary Material). These three sections requested information on: general flock management, history of parasitic infections, treatment against GINs and *F. hepatica*, timing, frequency, and purpose of treatment, background, the farmers’ opinions regarding efficacy of treatment and whether they experienced parasites as an increasing problem. All questions were mandatory. The questions were designed as multiple choice, of which 15 offered the possibility to select several alternatives. Additionally, one ranking question was included.

### Personal data

In order to obtain identifiable information, such as contact information of the respondents, a notification form for personal data was submitted to NSD (Norwegian Centre for Research Data) prior to the collection of the data. All recipients of the questionnaire provided consent regarding this according to the EU GDPR (General Data Protection Regulation) before participating in the questionnaire.

### Statistical methods

Data management and statistical analysis was performed using Excel (Microsoft Office 365 ProPlus) and Stata SE/16.0 (Stata Statistics/Data Analysis: Release 16. College Station, TX: StataCorp LLC). Descriptive analysis was carried out for each variable to generate frequencies for categorical variables and means with standard deviations for continuous variables. Associations between categorical variables were investigated by using contingency table analysis (Fisher’s exact test). Pearson correlation coefficient was used for continuous data. For calculations of significance based on frequencies (obtained from categorical variables), contingency table analyses (Pearson chi square test) were used. A *p*-value < 0.05 was regarded as significant.

## Results

**Figure 1:**
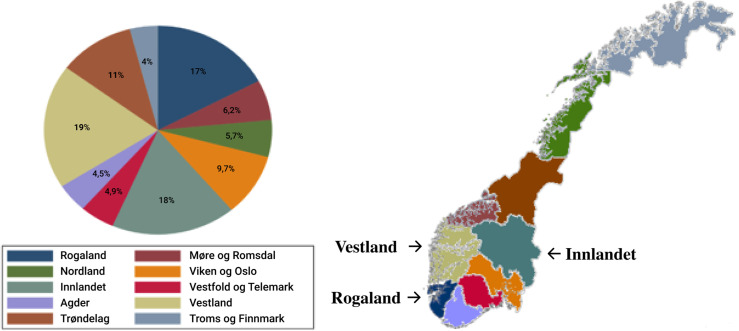
Geographical distribution of respondents displayed by piechart and map of Norway. Oslo and Viken counties are merged, as there was only 1 participant from Oslo.

A total of 1378 sheep farmers responded to the questionnaire survey, resulting in a response rate of 25%. The respondents were located in all 11 counties of Norway, with farmers from Vestland (19%), Innlandet (18%), and Rogaland counties (17%) representing the highest proportions of the respondents (Fig. 1). These three counties have the highest sheep populations in Norway and the number of respondents in each county also correlated strongly (*r* = 0.90) with the general flock distribution in Norway [43]. All sheep flocks were included in the analysis, regardless of flock size. These flocks represented 126,772 winter-fed sheep, corresponding to approximately 13% of the total sheep population in Norway [44].

### General information

Mean flock size was 92 winter-fed sheep with a range of 3–750 (Table 1). In total, the dominating breeds were the Norwegian white sheep (norsk kvit sau – 70%), the Old Norwegian short tail (spælsau – 13%), and the Old Norwegian sheep (villsau – 6%). Other breeds (10%) included a mixture of breeds and crosses thereof (data not shown). May was the most common month of turn out onto pasture, while the most frequent month of turning in (housing) was October. The majority of flocks had access to wet and moist areas on pasture (90%), and 43% of the flocks had access to pasture during the autumn/winter period. Most flocks were housed on slatted floors (60%). Approximately half of the respondents raised only sheep (49%). For those having mixed livestock on their farms, cattle were the most frequently reported additional livestock species (27%) (Table 1).

**Table 1:** Descriptive data of Norwegian sheep flocks in the present survey (*n* = 1378).

	Mean	Range	95% CI
Flock size	92.0	3–750	87.8–96.2
Most common month of turnout	May		
Most common month of housing	October		
	*n*	%	95% CI
Organic farming	154	11.2	9.6–13.0
Access to outdoor areas during indoor period	590	42.8	40.2–45.5
Access to wet/moist areas on pasture	1237	89.8	88.1–91.3
Type of floor at housing			
Slatted floor	832	60.4	55.7–63.0
Solid floor	377	27.4	25.0–29.8
Combination	169	12.3	10.6–14.1
Total	1378	100	
Mixed livestock at farm*			
Only sheep	675	49.8	46.3–51.7
Cattle	366	26.6	24.2–29.0
Horses	254	18.4	16.4–20.6
Goats	63	4.6	3.5–5.8
Other	220	16.0	14.1–18.0

*Possible to select several alternatives, thus total percentage exceeds 100%.

### Parasite control practices

**Figure 2:**
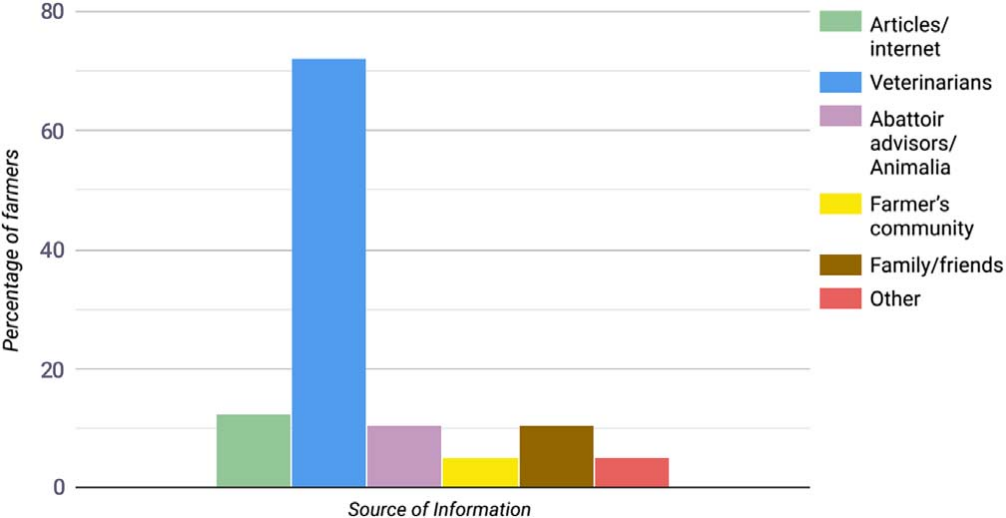
Most important advisor for parasite treatment perceived by the farmers (members of NSRS, Animalia). Possible to select several alternatives, thus total percentage exceeds 100.

In total, 89% of the farmers were satisfied by the guidance received by their veterinarian concerning parasite treatment. Most farmers (79%) were in contact with their/a veterinarian 1–2 times a year regarding parasite control. The veterinarian was regarded as the most important advisor for treatment of parasites by 72% of the respondents. Between 5 and 12% also reported other sources of information as being most important (Fig. 2). The main factors for deciding when to treat against helminths were previous experience (70%), and at housing (64%), while only 11% of the farmers used parasitological analysis of fecal samples as an indicator for treatment. Over 60% stated that fecal samples had never been submitted for analysis for parasites (Table 2). For dose estimation of anthelmintics, most farmers weighed a medium-sized animal and administered the drug to the whole flock based on this (36%). However, 14% estimated the dose based only on visual appraisal of sheep weight. The most common reason for checking the drench gun was if the farmer suspected that it was faulty (36%), whereas 8% never checked the accuracy of the equipment before administering the anthelmintics (Table 2). According to the responses, the use of BZ had been reduced from 2018 (52%) to 2019 (47%) (*p* < 0.01). On the other hand, the use of ML increased from 2017 (23%) to 2019 (36%) (*p* < 0.001). The combined use of BZ and ML slightly increased from 2017 (10%) to 2019 (11%) (*p* > 0.05) (Table 3). Over half of the farmers (63%) reported that bought-in stock were treated with anthelmintics after purchase, while fewer than half of the farmers (48%) reported using quarantine.

**Table 2:** Descriptive data of parasitological analysis (FEC = fecal egg count), timing of treatment, and dosage regime of the flocks included in this study (*n* = 1378).

		*n*	%	95% CI
Frequency of FEC per year*	Never	891	67.7	62.1–67.2
Lambs	1 time	138	10.0	8.5–11.7
	2 times	36	2.6	1.8–3.6
	3 or more	26	1.9	1.2–2.8
	On suspicion	428	31.1	28.6–33.6
Frequency of FEC per year*	Never	864	62.7	60.1–65.3
Ewes	1 time	148	10.7	9.2–12.5
	2 times	34	2.5	1.7–3.4
	3 or more	21	1.5	0.9–2.3
	On suspicion	414	30.0	27.6–32.5
Basis for timing of treatment*	Previous experience	959	69.6	67.1–72.0
	Housing	885	64.2	61.6–66.8
	Pasture rotation	497	36.1	33.5–38.7
	Regularity	350	25.4	23.1–27.8
	Clinical signs	333	24.2	21.9–26.5
	Weather	258	18.7	16.7–20.9
	FEC	155	11.2	9.6–13.1
Determination of dosage	Weigh each animal	1	0.1	0.0–0.4
	Visual estimation of weight	194	14.1	12.3–16.0
	Weighing a medium sized animal	492	35.7	33.2–38.3
	Weighing the largest animal	235	17.1	15.1–19.1
	Combination, not specified	456	33.1	30.6–35.6
	Total	1378		
Drench gun calibration per year	Never	115	8.4	6.9–9.9
	On suspicion that it doesn’t work	500	36.3	33.7–38.9
	Once	232	16.8	14.9–18.9
	1–2 times	284	20.6	18.5–22.8
	More often	247	17.9	15.9–20.1
	Total	1378		

*Possible to select several alternatives.

**Table 3:** Anthelmintics against GINs used during 2017–2019, according to the farmer responses (*n* = 1378): Benzimidazoles (BZ), Macrocyclic lactones (ML), and combination of both anthelmintic classes (BZ + ML).

Anthelmintic	2017*			2018			2019		
	*n*	%	95% CI	*n*	%	95% CI	*n*	%	95% CI
BZ	718	52.3	49.6–54.9	722	52.4	49.7–55.1	650	47.2	44.5–49.8
ML	313	22.8	20.6–25.1	416	30.2	27.8–32.7	497	36.1	33.5–38.7
BZ + ML	141	10.3	8.7–12.0	148	10.7	9.2–12.5	154	11.2	9.6–13.0

*n = 1374.

### Anthelmintic treatment

#### Nematodes

**Figure 3:**
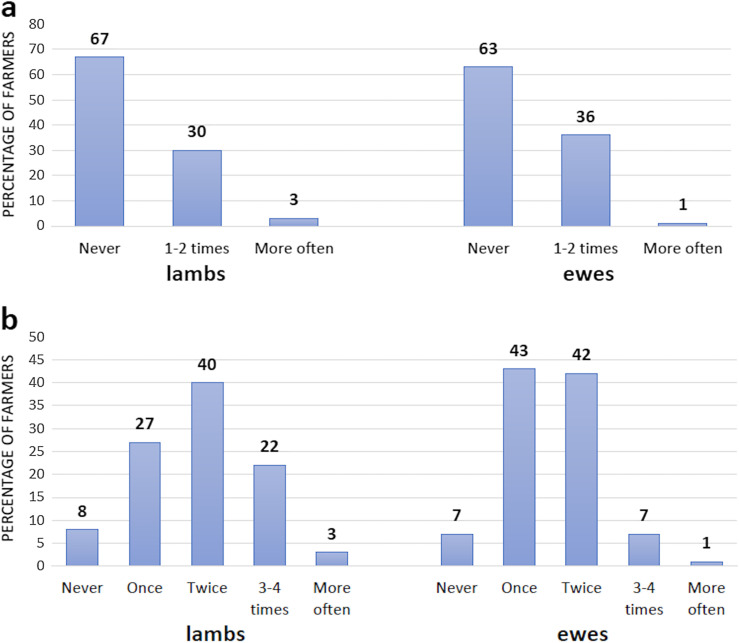
Frequency of treatment against (a) GINs in lambs (*n* = 1266) and ewes (*n* = 1262), and (b) *F. hepatica* in lambs (*n* = 1270) and ewes (*n* = 1293) during 1 year in these flocks. Incomplete/ambiguous responses were excluded, thus the n value (number of respondents) varies. Percentages are indicated above the bars.

On average, lambs were drenched twice during a year. A quarter of the farmers (25%) treated their lambs more often (Fig. 3), of which 62% of them were located on the west coast of Norway (Rogaland and Vestland). Adult sheep were drenched 1.5 times, on average, per year. Few farmers (8%) treated adult sheep more than twice a year, of which 53% were located on the west coast of Norway (Rogaland and Vestland). When asked about the purpose of treatment, 80% of the farmers stated that they treated prophylactically (preventative), either without having experienced any previous problems with GINs (53%), or following previous GIN-related issues (27%), while only 10% of them treated therapeutically (i.e., due to symptoms/disease).

#### Fasciola hepatica

Over half of the respondents never treated their lambs (67%) or adult sheep (63%) against flukes. Of those treating, a frequency of 1–2 times a year was most common in lambs (30%) and adult sheep (36%) (Fig. 3). More than half of those reporting a higher treatment frequency (>2 times a year) in lambs (58%) and adult sheep (69%) were located in Rogaland county. Among those treating against *F. hepatica*, ABZ (Valbazen^®^) was the main drug used (70%), and secondly TCBZ (Fasinex^®^) (27%), while 3% reported other non-specified drugs. In total, 24% treated prophylactically, of which approximately 10% did so due to previous problems with this parasite. Furthermore, 11% treated based on information on liver condemnation at the abattoir, while 8% treated due to clinical signs/disease (Table 4). About a quarter of the flocks (25%) had a history of condemned livers at the abattoir. There was a significant association (*p* < 0.001) between liver condemnation and access to moist and wet areas on pasture. In contrast, no correlation with history of condemned liver and access to pasture during autumn and winter was obtained. Furthermore, condemned liver was associated (*p* < 0.001) with having cattle as an additional livestock. In contrast, no correlation was found with keeping other livestock, such as goats and horses (Table 5).

**Table 4:** Purpose of anthelmintic treatment against nematodes and *F. hepatica* (*n* = 1378).

	*n*	%	95% CI
Nematodes			
Prophylactic – no previous problem with GINs	733	53.2	50.5–55.9
Prophylactic – previous problem with GINs	374	27.1	24.8–29.6
Therapeutic	135	9.8	8.3–11.5
No treatment	136	9.9	8.3–11.6
Total	1378		
*F. hepatica*			
Prophylactic – no previous problem with *F. hepatica*	186	13.5	11.7–15.4
Prophylactic – previous problem with *F. hepatica*	145	10.5	9.0–12.3
Therapeutic	108	7.8	6.5–9.4
Liver condemnation	146	10.6	9.0–12.3
No treatment	793	57.6	54.9–60.2
Total	1378		

**Table 5:** Association between condemned liver reported by farmers and management factors (*n* = 1378).

	Liver condemnation
Cattle at farm	***
Horse at farm	n.s.
Goat at farm	n.s.
Wet areas on pasture	***
Out during housing-period	n.s.

***p < 0.001, n.s. = not significant.

### Farmers’ perception of parasites

When asking the farmers which parasites were present in their flocks, considerable uncertainty was apparent, with between 44% and 72% of farmers reporting uncertainty regarding parasite species. The parasite associated with most uncertainty was *H. contortus* (72%). According to the farmers’ responses, *F. hepatica* and *H. contortus* were present in only 17% and 8% of the flocks, respectively (data not shown). Nevertheless, 11% reported that helminths were an increasing problem in their flocks, of which 50% were located at the west coast of Norway (Rogaland and Vestland). Furthermore, 10% answered that they suspected poor anthelmintic efficacy, of which approximately half (49%) were also located on the west coast.

## Discussion

In the present study, we report on: (i) management practices against major helminth pathogens in Norwegian sheep flocks, and (ii) the farmers’ perceptions regarding helminths and anthelmintic efficacy. Furthermore, we compare our findings with those of the previous questionnaire survey regarding worm control practice among Norwegian sheep farmers [14]. However, due to differences in study design, distribution, and respondents, between-study comparisons are somewhat limited.

The mean treatment frequencies of lambs (2.0) and ewes (1.5) against GINs was slightly lower than previously found in Norway [14]. Similar drenching rates for GINs have been reported on sheep farms in Sweden, Denmark, and the Netherlands [23, 29, 37], while it appears to be somewhat lower than in other European countries, such as Germany, Great Britain, and Ireland [3, 12, 30]. Farmers in the west coast area of Norway reported the highest frequency of treatment against GINs and *F. hepatica.* Similar findings were presented in the survey of 2007, where the drenching frequency in lambs and ewes was greater in the coastal area than in inland and northern areas [14]. This probably reflects the higher prevalence of both GINs and *F. hepatica* in this region [15], which may be explained by the milder climate with more rainfall, resulting in an environment that supports development, survival and transmission of these parasites. However, the participants in our study reported a high level of uncertainty regarding which parasites were present in their flock, with unknown status most frequently reported for *H. contortus* (72%). This probably reflects that few farmers (15%) regularly tested their animals for parasites. Furthermore, this emphasizes the importance of informed treatment, to avoid possible excessive drenching when the presence of helminths in their flock is unknown. Additionally, low infection awareness has been identified to be an important barrier for adoption of sustainable GIN control [46]. About a quarter of the farmers had experienced condemned liver at the slaughterhouse, but only 17% reported that *F. hepatica* were present in their flock. The significant association (*p* < 0.001) detected between a history of condemned liver and having access to wet and moist areas on pasture is an expected result, as the lifecycle of liver fluke depends on specific species of aquatic snails as intermediate hosts [4, 34]. The lack of correlation between a history of condemned liver and access to pasture during autumn and winter could be associated with the Norwegian climate, with cold temperatures delaying the development of the parasite on pasture [20]. The significant association (*p* < 0.001) detected between condemned liver and keeping cattle as an additional livestock could be explained by *F. hepatica* being a generalist infecting several hosts [25], and moreover, be related to pastures suitable for both cattle and sheep.

Treatment on fixed occasions (i.e., turn out onto pasture, turning in/housing, previous experience/routines), rather than using parasitological analysis as a tool to assess the timing or efficacy of treatment, appears to be a common feature among Norwegian sheep farmers, similar to reports from several European countries [11, 32, 37]. The reasons for this are likely to be multifactorial. The value of diagnostic testing might be underestimated by farmers, if they experience acceptable results following treatment based on their own routines. Farmers may also consider diagnostic testing as an unnecessary expense, especially if the FEC-results do not provide a clear indication for further action. Farming involves many different challenges, such as time management, ensuring the health and welfare of stock in general, maintenance tasks, compliance with regulations, consumers’ demands, etc. Taking these into account, it is likely that other farm-related practical challenges might be considered as higher priorities by the farmer than parasitological testing, especially when anthelmintic treatment is regarded as non-problematic. Most farmers (90%) in this study did not suspect lack of efficacy of the treatment, and therefore altering their already established routines might not be perceived as necessary. Another potential factor could be lack of encouragement from veterinarians regarding parasitological analysis. In the previous study among Norwegian sheep farmers, none of them reported FEC as an indicator for treatment [14]. Combined, these findings suggest that informed treatment against important helminth infections in Norwegian sheep is still poorly adopted.

Although parasitological analysis seems to be of low priority among the farmers in general, most of the respondents (79%) were in contact with their veterinarian 1–2 times a year specifically concerning parasite control. This is probably connected with the prescription-only status of anthelmintics in Norway, but could also be related to guidance from the veterinarian. Either way, this should be seen by veterinarians as an opportunity to give evidence-based advice and encourage sustainable parasite control.

The previous study by Domke revealed that most of the sheep farmers (79%) estimated the appropriate anthelminthic dose based on visual appraisal of sheep weight and almost a third (27%) never checked the accuracy of the drench gun [14]. The importance of calibration of equipment, avoiding under-dosing, and correct administration of anthelmintics has been an area of focus in the Sheep Health Service in Norway following the previous findings [1, 21, 22]. Thus, it seems as though the situation has improved in recent years, with visual appraisal apparently less frequently used (14%) and with more farmers (92%) now calibrating their drench guns more often.

The use of BZ was slightly reduced from 2018 to 2019. In contrast, use of ML increased substantially from 2017 to 2019. The same trend was already detected in the period 2005 to 2007 [14] and seems to be continuing. This could suggest that ML are preferred, and that BZ are considered to be less effective. However, this statement has to be further elucidated, as there may be other influencing factors such as, for instance, marketing.

Quarantine routines have been highlighted as an important factor to support a sustainable parasite control regime and to prevent the introduction of AR [32, 37]. Data concerning the proportion of farmers that bought-in sheep was not obtained in this survey. However, more farmers seem to simply drench newly purchased animals (63%) than performing true quarantine (48%). Thus, this indicates that there is the potential to increase quarantine practices among Norwegian sheep farmers.

The use of prophylactic anthelmintic treatment against GINs was widespread among the farmers in this study and seems to be a relatively common practice regarding *F. hepatica* as well. The latter finding, combined with broad spectrum ABZ being the preferred drug against flukes, suggests that GINs are exposed to unnecessary selection pressure. The proportion of farmers that specifically used a TT or TST strategy was not investigated in this survey. However, the finding that prophylactic treatment at fixed occasions is the most common practice, indicates that blanket treatment of the whole flock is the main approach used. Farmers’ attitudes have been found to be an essential influencing factor to adoption of informed treatment [47]. Another study found that the willingness of farmers to implement TST was strongly associated with availability of guidance and clinical markers. Additionally, the reduced costs by minimizing use of anthelmintics was an appealing factor [5]. Based on the finding that most farmers (72%) largely trust the veterinarian regarding parasite control measures, this appear to be a promising route to communicate the beneficial aspects that follow implementation of a sustainable control strategy, and thereby highlights the potential of the veterinarian’s future role.

In general, most farmers in this study did not suspect poor anthelmintic efficacy, which is similarly to previous reports from Belgium, Great Britain, and Ireland [11, 32]. Nevertheless, the study among Belgian sheep farmers showed that most of them perceived anthelmintic efficacy as good, despite reduced efficacy being detected by FECRT [11]. The perception of having increased problems related to helminths and/or poor anthelmintic efficacy was more common in Rogaland and Vestland counties (west coast) compared to other counties. This is substantiated by the higher treatment frequency observed in this area. In the study performed between 2008 and 2009 by Domke et al. [13], AR in GINs against BZ was 10.5% in randomly selected sheep flocks, but 31.0% in potential risk flocks, selected based on high frequency drenching, use of dose-and-move strategy, and intensive grazing of home pastures. The latter flocks were all located on the west coast. Although our findings are based on subjective impressions and cannot be used to gauge the occurrence of AR, they provide an insight into the farmers’ perceptions of their own risk under current conditions.

The questionnaire survey was distributed to farmers throughout Norway, and the responses obtained represent flocks in all counties. Thus, although three counties (Vestland, Innlandet and Rogaland) comprise the highest proportions of respondents, they also represent the highest sheep populations in Norway. The strong correlation between number of respondents and geographical distribution of sheep farmers suggests that regional bias is reduced. However, the mean flock size for members of NSRS is 82 sheep (> 1 year) [2], but 65.5 sheep (> 1 year) for the total sheep holdings in Norway [45]. This may indicate a sampling bias (e.g., by respondents having a higher stocking rate and thereby a potentially higher treatment frequency).

A non-response bias is present if the response-rate is limited due to the survey topic [36]. If the farmers that did not respond have little interest in this topic, a possible explanation could be a perception of parasites as being insignificant in their flock. This could, in turn, influence the results. Nevertheless, the majority of the respondents did not perceive gastrointestinal helminths as an increasing problem (89%) and did not suspect poor anthelmintic efficacy.

Collection of data by this method can generate response-bias, as the answers are subjective and cannot be controlled. This emphasizes the importance of designing clear, non-leading questions that reduce the chance of misinterpretation [24]. Pre-testing the survey by a pilot study is a method that can be used to improve the design of the questions [48]. After conducting our pilot study on a limited number of farmers, some questions were edited to make them clearer for the participants, thereby minimizing possible response-bias.

## Conclusion

The prophylactic use of anthelmintics seem still to be a common and widespread strategy among Norwegian sheep farmers. Informed treatment decisions, based on the results of parasitological analysis, are largely lacking. However, our data indicate that there may be increased awareness among farmers regarding correct administration of anthelmintics. According to our results, the use of BZ seems to be declining, while use of ML is increasing. The present study provides up-to-date information on the treatment practices of helminths in sheep in Norway and puts the results in a Europe-wide context.

## Supplementary Material

The supplementary material of this article is available at https://www.parasite-journal.org/10.1051/parasite/2021061/olm. Questionnaire regarding gastrointestinal parasites in sheep in Norway.

## Conflict of interest

There are no conflicts of interest.
