# Supplementary material for: Treatment against helminths in Norwegian sheep: a questionnaire-based survey
Source: Parasite. 2021 Sep 1;28:63. doi: 10.1051/parasite/2021061 (PMC10649840; doi:10.1051/parasite/2021061)
Supplement: Supplementary file 1 — Questionnaire regarding gastrointestinal parasites in sheep in Norway. [file parasite-28-63-s1.pdf]

Treatment against helminths in Norwegian sheep: a questionnaire-based survey.

Maiken Gravdal, Lucy Robertson, Kristoffer R. Tysnes, Johan Höglund, Christophe Chartier, Snorre Stuen

Corresponding author: Maiken Gravdal, Norwegian University of Life Sciences, Faculty of Veterinary Medicine, Department of Production Animal Clinical Sciences, Svebastadveien 110-112, 4325 Sandnes, Norway

maiken.gravdal@nmbu.no

Questionnaire regarding gastrointestinal parasites in sheep in Norway.

Dear sheep farmer!

This questionnaire is part of a four-year project to investigate gastrointestinal parasites in sheep. It is a multinational and multi-institutional project led by NMBU, Faculty of Veterinary Medicine and in collaboration with the Health Service for Sheep – Animalia.

You can answer the questionnaire here (link). The questionnaire should take 15 minutes to complete. Everyone completing the questionnaire will be entered into a draw with the possibility of winning a tablet computer or a one-year membership of the Norwegian Sheep Recording System (winner chooses).

Nematodes and liver flukes are widespread and are an important cause of clinical disease and production losses in sheep. The background of this project is that a lack of effect (resistance) has been found for drugs used to treat and prevent gastro-intestinal parasites in sheep in Norway. This leads to challenges related to the practical handling of infection, especially regarding the nematode *Haemonchus contortus* and the common liver fluke, *Fasciola hepatica*. Therefore, we want to investigate the prevalence of different parasites, effects of treatment, and the consequences they may have on production.

Based on the information obtained from this questionnaire, we would like to invite some farmers to participate in a sampling program during spring 2020/2021, to assess the efficacy of anthelmintics. Fecal samples from sheep will be collected from different parts of the country. This is a good opportunity to check the parasite-burden and the effect of anthelmintic treatment in your own flock.

The data will not be anonymous to us as we will see the questionnaire results, but all answers will be handled confidentially, and all results will be anonymized when published. Results from the questionnaire will be connected to production data in the Norwegian Sheep Recording System, in agreement with Animalia.

Thank you for your participation!

Best regards,

Maiken Gravdal

Ph.D.-student

Questionnaire regarding parasites in sheep in Norway.

**Name:**	**Farm number:**
**Street name:**	**Zip code and place:**
**Municipality:**	**County:**
**Phone number:**	**E-mail:**

General information on the holding.

S1

**How often are you in contact with the veterinarian regarding parasite control in one year?** *Check a box*
0 ☐ 1-2 ☐ 3 or more ☐

S2

**Do you use organic farming?** *Check a box*
yes ☐ no ☐ partly ☐

S3

**Which breed(s) of sheep do you keep?** *Specify number of winter-fed sheep*	**Number (winter-fed)**
Norwegian White Sheep	
Old Norwegian Sheep	
Old Norwegian Short Tail	
Texel:	
Other:	
**Total number of winter-fed sheep**	

S4

Do you have other livestock at your farm? *Check box(es)*

Cattle ☐

Horses ☐

Goats ☐

Other (specify):…………………………… ☐

No ☐

S5

What type of flooring do your sheep have during winter/housing period? *Check a box*

Slatted floor (expanded metal, plastic, wood, composite) ☐

Solid floor with straw/wood shavings ☐

Both solid and slatted floor ☐

S6

Which month were the sheep turned out onto pasture? *Check box(es)*

	2018	2019
April	☐	☐
May	☐	☐
June	☐	☐
Other (specify):…………………………………	☐	☐

S7

Which month were the sheep housed in the winter/autumn? *Check box(es)*

	2018	2019
October	☐	☐
November	☐	☐
December	☐	☐
Other (specify):…………………………………	☐	☐

S8

Is it possible for the animals to go out onto pasture during autumn/winter after housing (e.g., during fine weather)? *Check a box*

Yes ☐ No ☐

S9

What kind of pasture do the sheep use during the different seasons? *Check box(es)*

	Spring	Summer	Autumn	Winter
Cultivated pasture	☐	☐	☐	☐
Home pasture	☐	☐	☐	☐
Rangeland	☐	☐	☐	☐
Forest pasture	☐	☐	☐	☐
Other (specify):…………………………..	☐	☐	☐	☐

S10

Do the sheep have access to wet/moist areas (e.g. bogs/marshland, ditches, etc.) when on pasture?

Check a box Yes ☐ No ☐

S11

What kind of water source do the animals have access to on pasture? *Check box(es)*

Surface water (e.g., natural ponds, streams, lakes)	Yes ☐	No ☐
Trough with surface water	Yes ☐	No ☐
Trough with treated water	Yes ☐	No ☐

S12

Which other species use the same pasture together with your sheep (mixed grazing)? *Check box(es)*

	**Home pasture**	**Rangeland**
No mixed grazing	☐	☐
Goats	☐	☐
Cattle	☐	☐
Wild deer	☐	☐
Other (specify):………………………………	☐	☐

S13

If you have mixed grazing, are the other species treated against parasites?

Check a box Yes ☐ No ☐ Unknown ☐ Not applicable ☐

S14

Are newly purchased animals treated with anthelmintics before being introduced to your flock?
Check a box

**Before purchase**	Yes ☐	No ☐	Unknown ☐
**After purchase**	Yes ☐	No ☐	Unknown ☐

Is any form of quarantine (housed/kept separately etc.) used in conjunction with purchase of animals? *Check a box*

Yes ☐ No ☐ Unknown ☐

S15

Have you seen or experienced any of the following in your flock during the last 5 years?
Check a box

	Yes	No	Unknown
Pale/anemic animals	☐	☐	☐
Diarrhea	☐	☐	☐
Bottlejaw	☐	☐	☐
Loss of wool on pasture	☐	☐	☐
Reduced growth on pasture	☐	☐	☐
Sudden death on pasture	☐	☐	☐
Coughing	☐	☐	☐
Condemned liver (at abattoir)	☐	☐	☐
Low slaughter weight	☐	☐	☐

S16

How often are parasitological analyses performed in your flock (fecal samples with egg count)? *Check a box*

	Lambs	Adults
Never	☐	☐
Once a year	☐	☐
Twice a year	☐	☐
3 or more times per year	☐	☐
On suspicion (diarrhea, other symptoms)	☐	☐

S17

What are the reason(s) for checking for parasites in your flock? *Check box(es)*

Disease/death ☐

Diarrhea ☐

Reduced growth ☐

General surveillance ☐

Other ☐

S18

Who do you consider to be your most important advisor regarding treatment against parasites, graded from 1 to 5, where 1 is the most important and 5 is the least important advisor?

Check a box

	1	2	3	4	5
Journals/articles/internet	☐	☐	☐	☐	☐
Veterinarian	☐	☐	☐	☐	☐
Abattoir advisors/Animalia	☐	☐	☐	☐	☐
Farmers community	☐	☐	☐	☐	☐
Neighbors/colleagues/family	☐	☐	☐	☐	☐
Other (specify):…………………………..	☐	☐	☐	☐	☐

S19

Which parasites do you have in your flock? *Check box(es)*

	**Present**	**Detected by egg count**	**Not present**	**Unknown**
Nematodirus battus	☐	☐	☐	☐
Haemonchus contortus	☐	☐	☐	☐
Tapeworm	☐	☐	☐	☐
Lungworm	☐	☐	☐	☐
Common liver fluke	☐	☐	☐	☐
Coccidia	☐	☐	☐	☐
Ticks	☐		☐	☐
Lice	☐		☐	☐

Treatment of gastrointestinal parasites and liver flukes:

S20

How do you decide the timing of treatment against parasites in your flock? *Check box(es)*

Experience from previous years	☐
Weather and climate	☐
Parasitological analysis (egg count)	☐
Pasture rotation	☐
Regularity, e.g. lambs every 3-4 week	☐
Disease (death/diarrhea/reduced weight gain)	☐
Housing for the winter	☐
Other, specify?...	☐

S21

Which anthelmintic preparation(s) have you used in the following years? *Check box(es)*

	2017	2018	2019
Valbazen vet	☐	☐	☐
Curaverm vet	☐	☐	☐
Panacur vet	☐	☐	☐
Ivomec vet	☐	☐	☐
Ivermax vet	☐	☐	☐
Dectomax vet	☐	☐	☐
Fasinex vet	☐	☐	☐
Cestocur vet	☐	☐	☐
Other (specify?):……………………………	☐	☐	☐

S22

How do you estimate the dose? *Check a box*

Individual weighing	☐
Visual appraisal	☐
Weigh medium-sized animal, and dose the rest based on this	☐
Weigh heaviest animal, and dose the rest based on this	☐
Other, specify?..	☐

S23

How often do you calibrate your drench gun during a year? *Check a box*

Never	☐
Only on suspicion that it is not working	☐
1 time	☐
1-2 times	☐
More often	☐

S24

Tick the appropriate boxes.

	Yes	No
Do you suspect a lack of effect from an anthelmintic drug?	☐	☐
Can you change spring pasture from one year to another?	☐	☐
Can you use a spring pasture that wasn't used during the previous autumn?	☐	☐
Have parasites in sheep been an increasing problem in your flock?	☐	☐
Are you satisfied by the guidance (*PT) you receive from the veterinarian?	☐	☐
Do you use preventive measures to reduce parasites on pasture:		
- Mowing/cutting of vegetation (after grazing)?	☐	☐
- Plowing?	☐	☐
- Drenching?	☐	☐

*PT = parasite treatment

Roundworm

S25

How often do you treat against roundworms (Valbazen, Curaverm, Panacur, Ivomec, Ivermax, Dectomax)? *Check a box*

	Lambs	Adults
Never	☐	☐
Once a year	☐	☐
Twice a year	☐	☐
3-4 times a year	☐	☐
More often	☐	☐

S26

When are adults treated against roundworms? *Check box(es)*

At housing ☐

At turnout to home pasture ☐

At turnout to rangeland ☐

Other, specify?......................... ☐

Not applicable ☐

S27

Why do you treat against roundworms? *Check a box*

Prophylactic – have not had previous problems with roundworms ☐

Prophylactic – have had previous problem with roundworms ☐

Treat when symptoms/disease observed ☐

Not applicable ☐

Haemonchus contortus

S28

If *Haemonchus* is present in your flock, when do you treat against it? *Check box(es)*

	Lambs	Adults
Treat in the autumn	☐	☐
Treat in the spring	☐	☐
3-4 times a year	☐	☐
More often	☐	☐

Not applicable ☐

S29

Which drug(s) do you use against *Haemonchus*? *Check box(es)*

Panacur/Valbazen/Curaverm ☐

Ivomac/Ivermax ☐

Other ☐

Not applicable ☐

S30

Do you have the impression that the treatment against *Haemonchus* is effective? *Check a box*

Yes	No	Not applicable
☐	☐	☐

Liver flukes

S31

How often do you treat against liver flukes (Fasinex, Valbazen)? *Check a box*

	Lambs	Adults
Never	☐	☐
1-2 times a year	☐	☐
More often	☐	☐

S32

Which drug(s) do you use against liver flukes? *Check box(es)*

Valbazen ☐

Fasinex ☐

Other ☐

Not applicable ☐

S33

Do you have the impression that the treatment against liver flukes is effective? *Check box*

Yes	No	Not applicable
☐	☐	☐

S34

Why do you treat against liver flukes? *Check a box*

Prophylactic – have not had previous problems with liver flukes ☐

Prophylactic – have had previous problems with liver flukes ☐

Treat when symptoms/disease observed ☐

Detected at abattoir ☐

Not applicable ☐

S35

Would you like to participate in this project?

Check a box Yes ☐ No ☐
